# Tetrahedral Framework Nucleic Acid Delivery of miR‐210‐3p Promotes Femoral Bone Regeneration Through EFNA3/AKT/STAT3‐Mediated Immunomodulation

**DOI:** 10.1111/cpr.70277

**Published:** 2026-09-01

**Authors:** Lei Hu, Kaixiao Xue, Jiahu Fang, Weitong Cui

**Affiliations:** ^1^ Department of Orthopaedics The First Affiliated Hospital of Nanjing Medical University Nanjing China; ^2^ Department of Orthopedics Sichuan Lansheng Brain Hospital and Shanghai Lansheng Brain Hospital Investment Co. Ltd. Chengdu China; ^3^ Psychiatric Laboratory and Mental Health Center, The State Key Laboratory of Biotherapy West China Hospital of Sichuan University Chengdu China

**Keywords:** AKT/STAT3 signalling, EFNA3, femoral critical defect, macrophage polarisation, miR‐210‐3p, tetrahedral framework nucleic acid

## Abstract

Persistent inflammation, insufficient vascularization and impaired osteogenesis limit the repair of critical‐sized femoral defects. Although microRNA‐210‐3p (miR‐210‐3p) can regulate these processes, its therapeutic use is constrained by poor extracellular stability and inefficient cellular entry. Here, miR‐210‐3p was incorporated into a tetrahedral framework nucleic acid (tFNA) to create tFNAs‐miR‐210‐3p. Dynamic light scattering, zeta‐potential analysis, atomic force microscopy (AFM) and capillary electrophoresis supported formation of the negatively charged nanosystem. In RAW264.7 macrophages, tFNA delivery increased Cy5‐labelled miR‐210‐3p‐positive cells from 1.24% for free miR‐210‐3p to 99.93%. Under lipopolysaccharide (LPS)‐induced inflammatory conditions, tFNAs‐miR‐210‐3p reduced CD86 and inducible nitric oxide synthase, increased CD206 and arginase‐1, enhanced endothelial tube formation and improved osteogenic differentiation. It suppressed M1 marker CD86/iNOS and elevated M2 marker CD206/Arg‐1 to resolve the inflammatory microenvironment. Mechanistically, miR‐210‐3p directly binds the 3′UTR of EFNA3 mRNA to inhibit its expression, thereby activating AKT/STAT3 signalling to switch macrophages toward reparative phenotype. In a rat critical‐sized femoral defect model, local tFNAs‐miR‐210‐3p treatment improved histological repair, macrophage polarisation, microcomputed‐tomography indices, vascularization and osteocalcin expression at 4 and 8 weeks. These findings identify tFNA‐mediated miR‐210‐3p delivery as a promising immunomodulatory strategy for vascularised bone regeneration.

## Graphical abstract

Schematic of tFNA‐miR‐210‐3p fabrication, macrophage regulation and femoral defect therapy: it enters macrophages, inhibits EFNA3 to induce M2 polarisation and boosts rat bone repair in vivo.

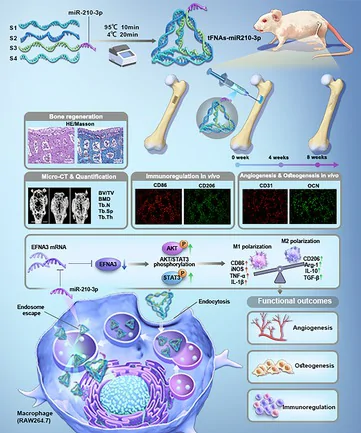

## 1 Introduction

Critical‐sized bone defects caused by trauma, infection, tumour resection or revision surgery remain difficult to treat. Successful repair requires coordinated resolution of inflammation, revascularization of the defect and osteogenic maturation. When the early inflammatory response persists, proinflammatory macrophage activity can inhibit endothelial network formation and mesenchymal stromal‐cell osteogenesis, thereby delaying or preventing structural restoration [1, 2].

Macrophages serve as central regulators of the osteoimmune microenvironment during bone regeneration. While transient M1 macrophage activation is required for early debris clearance, sustained M1 polarisation creates a chronic inflammatory niche that robustly suppresses both endothelial tube formation and mesenchymal stromal cell osteogenic differentiation, ultimately delaying or arresting bone defect repair. Persistently elevated secretion of pro‐inflammatory cytokines (TNF‐α, IL‐1β) from M1 macrophages disrupts core osteogenic transcription programs and impairs angiogenic signal transduction, highlighting the necessity of timely M1‐to‐M2 phenotypic switching to restore regenerative capacity [1, 3].

Contemporary nanomedicine increasingly combines rational material design with targeted and responsive delivery. Recent platforms span artificial‐intelligence‐guided carbon nanomaterials, vesicle‐assisted nanosystems, hypoxia‐responsive photosensitizers, catalytic nanozymes and biomimetic vesicles with bone‐marrow homing capability [4, 5, 6, 7, 8].

Beyond bone repair, framework‐nucleic‐acid systems have been reported to modulate inflammatory microenvironments in burn wounds, neuroinflammation, tendinopathy, sepsis, infected wounds and acute inflammatory arthritis, illustrating the broad immunoregulatory potential of programmable DNA nanomaterials [9, 10, 11, 12, 13, 14, 15].

miR‐210‐3p is a hypoxia‐responsive microRNA with reported roles in inflammation, angiogenesis and osteogenic regulation. Its direct therapeutic application, however, is limited by nuclease susceptibility and inefficient membrane crossing. Tetrahedral framework nucleic acids (tFNAs) are programmable DNA nanostructures assembled by sequence‐specific base pairing. Their defined geometry, biocompatibility and cellular uptake make them attractive carriers for nucleic‐acid therapeutics [16, 17].

Tetrahedral DNA systems have also been adapted as microRNA sponges and switchable microRNA‐therapeutic carriers, demonstrating that their programmable nucleic‐acid interfaces can support both inhibition and delivery of regulatory RNAs [18, 19].

Extensive protocols and application studies further demonstrate that tetrahedral and dynamic DNA frameworks can be functionalized for active targeting, responsive delivery and molecular imaging across diverse biomedical settings [20, 21, 22, 23, 24, 25].

Within regenerative medicine, framework nucleic acids have enhanced VEGF signalling, regulated macrophage‐centered cellular crosstalk, alleviated diabetic osteoporosis, supported infected‐bone‐defect repair and enabled mineralized immunomodulatory scaffolds for bone regeneration [26, 27, 28, 29, 30].

In this study, we engineered a tFNA bearing miR‐210‐3p and evaluated its physicochemical characteristics, macrophage uptake, immunomodulatory activity and effects on angiogenesis and osteogenesis. We further investigated the EFNA3/AKT/STAT3 axis and assessed therapeutic efficacy in a rat critical‐sized femoral defect model. We hypothesized that efficient tFNA‐mediated delivery of miR‐210‐3p would suppress EFNA3, promote AKT/STAT3 phosphorylation, favour reparative macrophage polarisation and thereby enhance vascularized bone regeneration.

## 2 Materials and Methods

### 2.1 Materials

Single‐stranded DNA components S1, S2, S3, S4 and the miR‐210‐3p‐modified strand were synthesized and purified by Sangon Biotech (Table S1). LPS, alkaline phosphatase (ALP) staining reagents and Alizarin Red S were obtained from commercial suppliers. RAW264.7 macrophages, human umbilical vein endothelial cells (HUVECs) and rat bone marrow mesenchymal stromal cells (BMSCs) were used for cellular experiments.

### 2.2 Assembly of tFNAs and tFNAs‐miR‐210‐3p

Equimolar component strands were mixed in TAE/Mg^2+^ buffer, heated to 95°C for 10 min and cooled to 4°C over 20 min to promote sequence‐programmed self‐assembly. Unmodified S3 was used for blank tFNAs, whereas the miR‐210‐3p‐bearing strand was used to assemble tFNAs‐miR‐210‐3p. Samples were stored at 4°C before use.

### 2.3 Physicochemical Characterization

Hydrodynamic size distributions and zeta potentials were measured by dynamic light scattering and electrophoretic light scattering, respectively. Morphology was examined by AFM, and capillary electrophoresis was used to compare the component strands, assembled tFNAs and tFNAs‐miR‐210‐3p.

### 2.4 Cell Treatment Groups

RAW264.7 macrophages were treated according to the five‐group design: (1) Control group: Normal culture medium without any treatment; (2) LPS model group: Cells incubated with 100 ng mL^−1^ LPS to establish inflammatory activation model; (3) LPS + tFNAs group: LPS stimulation plus blank tFNAs (250 nM); (4) LPS + free miR‐210‐3p group: LPS stimulation plus free miR‐210‐3p (250 nM); (5) LPS + tFNAs‐miR‐210‐3p group: LPS stimulation plus tFNAs‐miR‐210‐3p nanocomplex (250 nM). HUVECs and BMSCs were subsequently cultured with conditioned media collected from these macrophage groups. All interventions were synchronously administered for corresponding culture durations according to different functional detection requirements.

### 2.5 Cell Culture and CCK‐8 Assay

Cells were incubated at 37°C with 5% CO_2_. Grouping followed the unified scheme defined in Section 2.4. RAW264.7 macrophage biocompatibility was evaluated using the CCK‐8 assay and morphological observation after treatment with gradient LPS (0–200 ng mL^−1^) or tFNAs‐miR‐210‐3p (0–500 nM) over 0, 6, 12 and 24 h time points.

### 2.6 Cellular Uptake

Cy5‐labelled free miR‐210‐3p or tFNAs‐miR‐210‐3p was incubated with RAW264.7 macrophages for 6 h. Intracellular fluorescence was visualized by fluorescence microscopy (649/670 nm), and the percentage of Cy5‐positive cells was quantified by flow cytometry.

### 2.7 Angiogenesis, Osteogenic Differentiation and Cytokine ELISA Assays

HUVECs were cultured with conditioned supernatant harvested from five groups of RAW264.7 macrophages. Cell grouping was consistent with Section 2.4. HUVEC angiogenic activity was assessed via Matrigel tube‐formation assay and quantified by branch‐point number. BMSC osteogenic differentiation was evaluated by ALP staining and activity and by Alizarin Red S staining for matrix mineralization. For quantitative analysis of macrophage‐secreted soluble factors, identical macrophage culture supernatants from all five groups were harvested for enzyme‐linked immunosorbent assay (ELISA) using commercial kits in accordance with manufacturer protocols. Four representative cytokines were detected to reflect macrophage polarisation and regenerative bioactivity: interleukin‐1β (IL‐1β), a hallmark pro‐inflammatory mediator secreted by M1 macrophages; interleukin‐10 (IL‐10), a key anti‐inflammatory cytokine specific to reparative M2 macrophages; bone morphogenetic protein 2 (BMP‐2), a master regulator of osteogenic maturation; and vascular endothelial growth factor (VEGF), the primary driver of angiogenesis. Cytokine absorbance values were recorded spectrophotometrically. All ELISA measurements were performed in biological triplicate, and final cytokine concentrations were calculated against the standard curve supplied with each kit.

### 2.8 Macrophage Polarisation and EFNA3/AKT/STAT3 Analysis

Macrophage polarisation was evaluated by immunofluorescence and flow cytometry for CD86 and CD206. Western blotting was used to assess EFNA3 (Invitrogen, PA5‐101126, 1:500), phosphorylated and total AKT (abcam, ab81283, 1:5000), phosphorylated and total STAT3 (abcam, ab119352/ab32143, 1:5000/1:1000), iNOS (abcam, ab178945, 1:1000), arginase‐1 (abcam, ab96183, 1:500) and beta‐actin (abcam, ab8227, 1:1000).

### 2.9 Rat Critical‐Sized Femoral Defect Model

Fifty male adult Sprague–Dawley rats (200–220 g) were utilized for all in vivo animal experiments. Under general anaesthesia, a 2 mm full‐thickness critical‐sized defect was generated on the femoral diaphysis of each rat. Animals were randomly allocated to five experimental groups identical to the cellular grouping scheme defined above. After surgical intervention, rats received daily penicillin administration, were kept warm, and underwent routine wound monitoring. Corresponding therapeutic formulations were locally injected into the defect cavity of each rat according to group assignment. At predetermined time points (4 and 8 weeks post‐operation), all rats were humanely euthanized via intraperitoneal injection of excessive pentobarbital sodium at a dose of 150 mg kg^−1^ in accordance with standard animal welfare guidelines. After complete confirmation of cardiac arrest and disappearance of corneal reflexes, intact femoral specimens were harvested for subsequent microcomputed tomography (micro‐CT), histological staining and immunofluorescence analysis.

### 2.10 Micro‐CT, Histology and Tissue Immunofluorescence

Micro‐CT reconstructions were used to quantify bone mineral density (BMD), bone volume fraction (BV/TV), trabecular number (Tb.N), trabecular separation (Tb.Sp), trabecular thickness (Tb.Th), cortical tissue mineral density (Ct.TMD), cortical computed‐tomography value (Ct.CT) and cortical thickness (Ct.Th). Sections were stained with haematoxylin and eosin (H&E) and Masson's trichrome. Tissue immunofluorescence assessed F4/80 with CD86 or CD206 and evaluated CD31 and osteocalcin (OCN).

### 2.11 Statistical Analysis

Data are presented as mean ± standard deviation. Comparisons among multiple groups were performed using one‐way analysis of variance followed by Tukey's multiple‐comparison test. A two‐sided *p*‐value < 0.05 was considered significant (*n* = 10).

## 3 Results and Discussion

### 3.1 Formation and Characterization of tFNAs‐miR‐210‐3p

**FIGURE 1:**
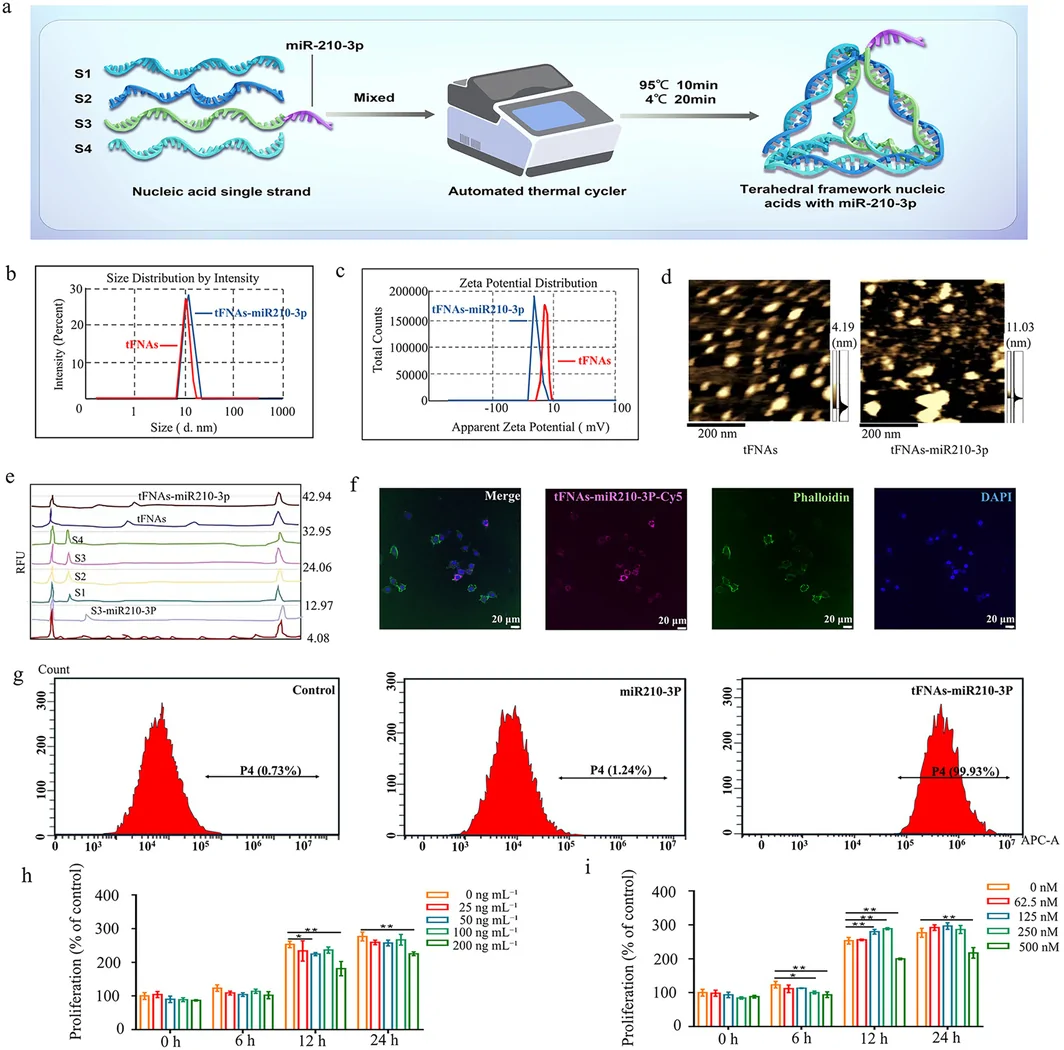
Assembly and physicochemical characterization of tFNAs and tFNAs‐miR‐210‐3p. (a) Assembly scheme (replace the current Chinese placeholder with the final schematic before submission). (b, c) Hydrodynamic‐size and zeta‐potential distributions of tFNAs and tFNAs‐miR‐210‐3p. (d) AFM images. (e) Capillary‐electrophoresis profiles of component strands and assembled products. (f, g) Fluorescence microscopy and flow‐cytometric quantification of cellular uptake. (h, i) CCK‐8 proliferation assays and representative bright‐field microscopy images showing the time‐ and dose‐dependent proliferative response of RAW264.7 macrophages treated with LPS (0–200 ng mL^−1^, a) or tFNAs‐miR‐210‐3p (0–500 nM, b) at 0, 6, 12 and 24 h.

The tFNA carrier and miR‐210‐3p‐bearing construct were assembled from sequence‐programmed nucleic‐acid strands (Figure 1a). Both formulations exhibited narrow nanoscale size distributions and negative surface potentials (Figure 1b,c). AFM images showed nanoscale structures for tFNAs and tFNAs‐miR‐210‐3p (Figure 1d), while capillary electrophoresis distinguished the component strands and assembled products (Figure 1e). Fluorescence microscopy confirmed cellular association of Cy5‐labelled tFNAs‐miR‐210‐3p (Figure 1f). Flow cytometry showed 0.73% Cy5‐positive cells in the control, 1.24% after exposure to free miR‐210‐3p and 99.93% after tFNAs‐miR‐210‐3p exposure (Figure 1g), demonstrating the delivery advantage of the tFNA carrier [17, 18]. Together, these measurements support successful formation of the miR‐210‐3p‐bearing framework. Quantitative values and replicate statistics should be reported to permit rigorous comparison [19, 20].

### 3.2 tFNA Delivery Enables Efficient Macrophage Uptake and Supports Angiogenic and Osteogenic Responses

**FIGURE 2:**
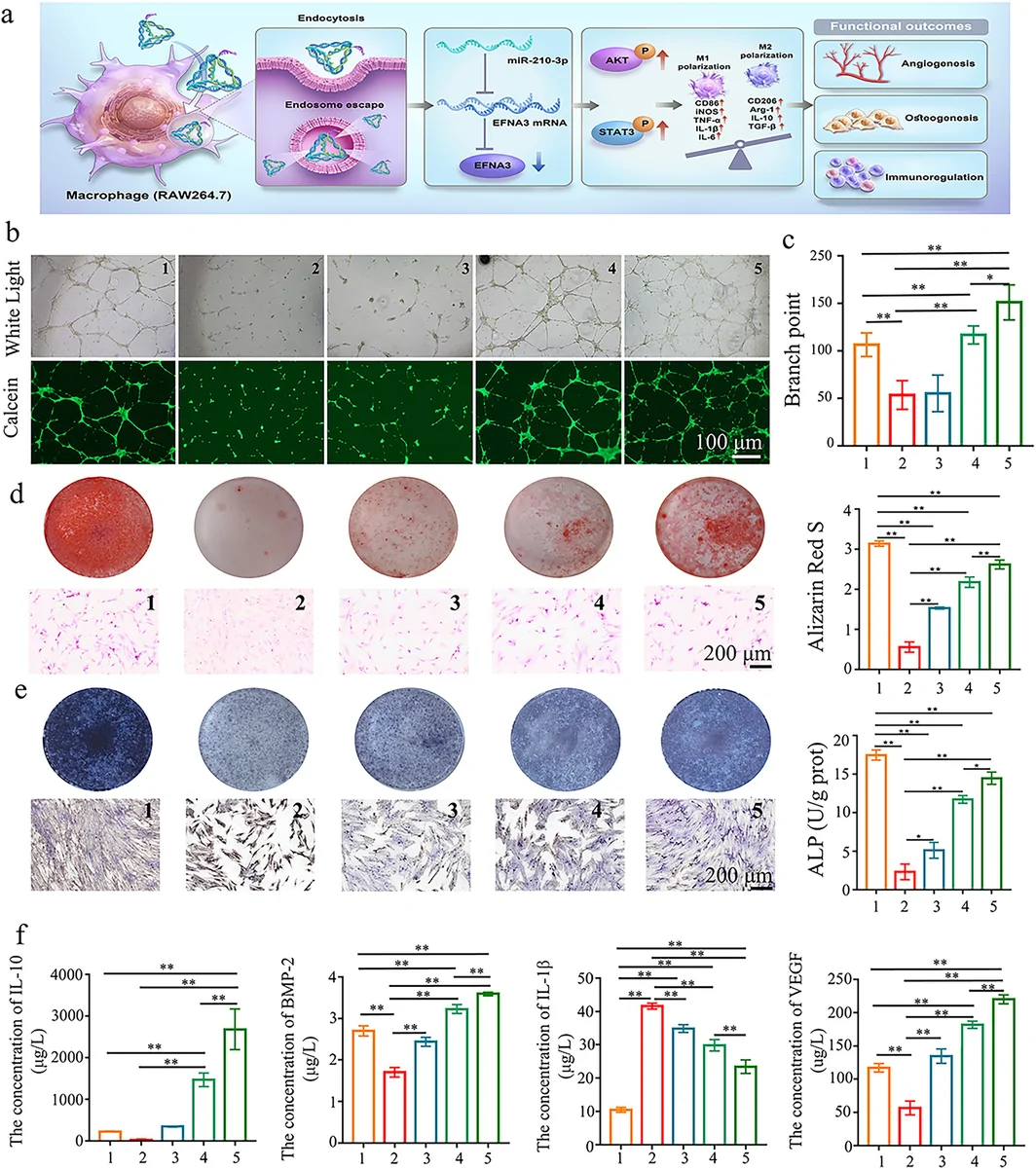
Effects on angiogenesis and osteogenic differentiation. (a) Proposed mechanism of tFNAs‐miR‐210‐3p‐mediated immunomodulation and regenerative activity. (b, c) HUVEC tube formation and branch‐point quantification. (d) Alizarin Red S staining. (e) ALP staining. (f) Cytokine ELISA assays (IL‐10, BMP‐2, IL‐1β and VEGF). Groups: 1, control; 2, LPS; 3, LPS + tFNAs; 4, LPS + miR‐210‐3p; 5, LPS + tFNAs‐miR‐210‐3p. *p < 0.05 and **p < 0.01; confirm *n* and statistical comparisons.

The proposed therapeutic sequence is efficient macrophage internalization of tFNAs‐miR‐210‐3p, suppression of EFNA3, activation of AKT/STAT3 signalling and a shift toward a reparative macrophage state that supports angiogenesis and osteogenesis (Figure 2a). Prior to subsequent functional experiments, optimal working concentrations of LPS and tFNAs‐miR‐210‐3p were screened via cell morphological observation and viability detection. Figure 1h showed that LPS ranging from 25 to 100 ng mL^−1^ exerted no significant cytotoxicity, while 200 ng mL^−1^ LPS significantly decreased cell viability after 12 h of incubation. Accordingly, 100 ng mL^−1^ LPS was selected to establish the cellular inflammatory model for subsequent experiments. Figure 1i revealed that 62.5–250 nM tFNA‐miR‐210‐3p maintained robust cell proliferation. In comparison, 500 nM tFNA‐miR‐210‐3p markedly suppressed cell viability at 12 h. Thus, 250 nM tFNA‐miR‐210‐3p was determined as the optimal working concentration with negligible cytotoxicity.

LPS markedly disrupted HUVEC network formation. Free miR‐210‐3p and particularly tFNAs‐miR‐210‐3p restored branch‐point formation, whereas blank tFNAs provided little improvement under the tested conditions (Figure 2b,c). Similarly, LPS reduced osteogenic staining, and tFNAs‐miR‐210‐3p produced the strongest recovery in ALP‐associated and mineralization readouts (Figure 2d,e). These findings demonstrate that tFNAs‑miR‑210‑3p exerts coordinated proangiogenic and pro‑osteogenic effects; nevertheless, the cross‑cell communication mechanism mediating such effects is not yet clarified in this study and warrants further exploration [25].

Consistent with the phenotypic shift of macrophages, ELISA quantification of culture supernatant cytokines further validated the regulatory function of tFNAs‐miR‐210‐3p on inflammatory, osteogenic and angiogenic secretory profiles (Figure 2f). The LPS‐stimulated Model group exhibited drastically elevated secretion of pro‐inflammatory IL‐1β, while the anti‐inflammatory reparative cytokine IL‐10 was markedly suppressed. Treatment with blank tFNAs or free miR‐210‐3p only partially reversed this inflammatory imbalance. In contrast, the tFNAs‐miR‐210‐3p group significantly downregulated IL‐1β release and induced the highest level of IL‐10 secretion, facilitating the transition from pro‐inflammatory M1 to anti‐inflammatory M2 macrophages.

For osteogenic and angiogenic functional factors, LPS treatment greatly reduced the production of BMP‐2 and VEGF. Single administration of tFNAs or miR‐210‐3p moderately recovered their expression, whereas combined delivery via tFNAs‐miR‐210‐3p achieved the most prominent upregulation of both BMP‐2 and VEGF. Collectively, the ELISA data verified that tFNAs‐miR‐210‐3p could simultaneously alleviate inflammatory stress, boost osteogenic BMP‐2 secretion, and promote angiogenic VEGF release, which synergistically facilitates bone repair by remodelling macrophage secretome toward a pro‐regenerative phenotype.

### 3.3 tFNAs‐miR‐210‐3p Promotes a Reparative Macrophage Phenotype Associated With EFNA3/AKT/STAT3 Signalling

**FIGURE 3:**
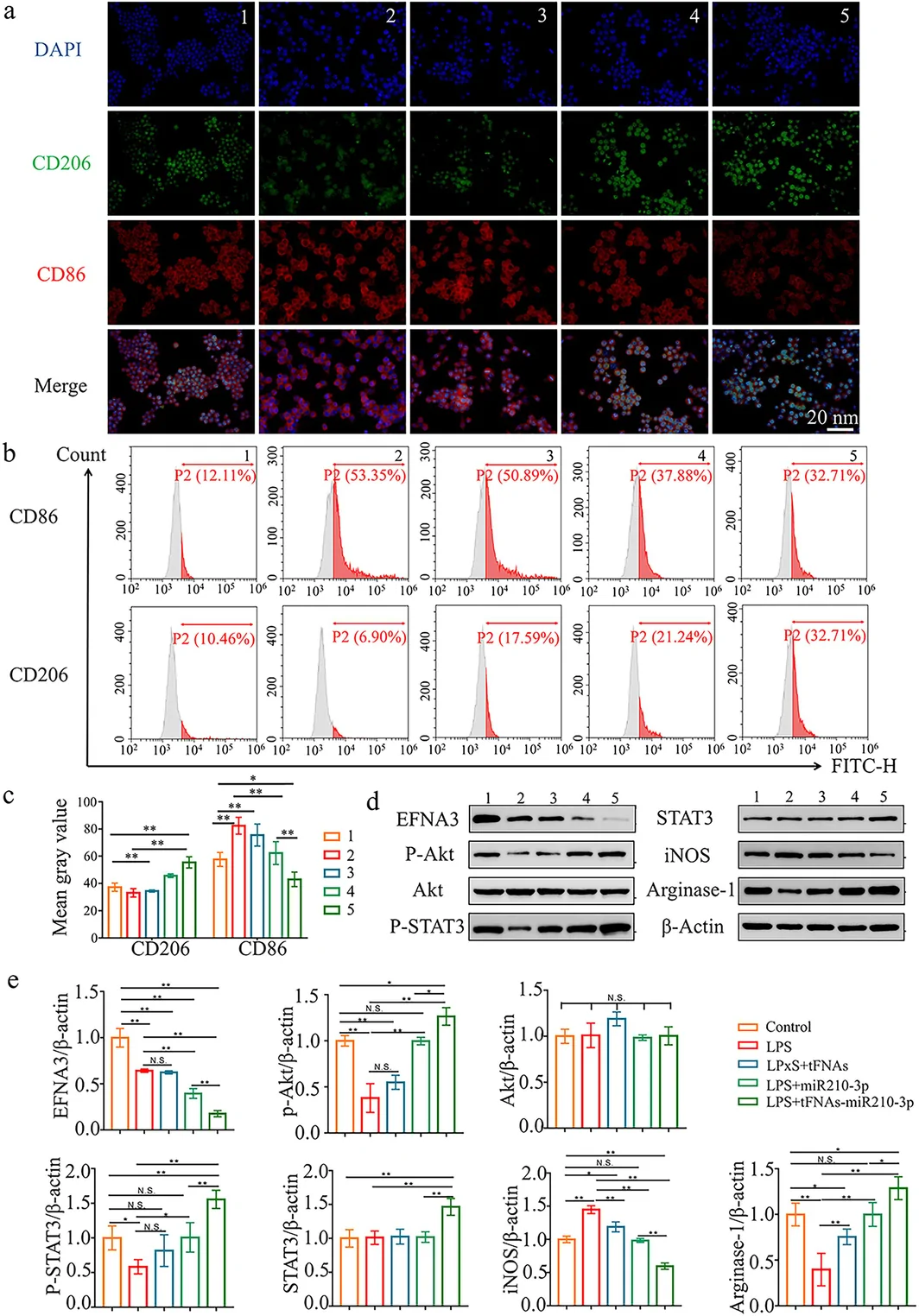
tFNAs‐miR‐210‐3p regulates macrophage polarisation and the EFNA3/AKT/STAT3 axis. (a) CD86 and CD206 immunofluorescence. (b) Flow‐cytometric analysis of CD86‐ and CD206‐positive cells. (c) Fluorescence‐intensity quantification. (d) Western blots for EFNA3, phosphorylated and total AKT, phosphorylated and total STAT3, iNOS, arginase‐1 and beta‐Actin. (e) Quantitative densitometric analysis of all proteins shown in (d). Groups are defined as in Figure 2. *p < 0.05 and **p < 0.01; confirm *n* and statistical comparisons.

Immunofluorescence, flow cytometry and fluorescence quantification showed that LPS increased CD86 and reduced CD206, whereas tFNAs‐miR‐210‐3p produced the most pronounced reversal of this polarisation pattern (Figure 3a–c). Flow cytometry showed CD86‐positive fractions of 12.11%, 53.35%, 50.89%, 37.88% and 32.71% and CD206‐positive fractions of 10.46%, 6.90%, 17.59%, 21.24% and 32.71% for Groups 1–5, respectively (Figure 3b) [11, 12, 13, 14, 26].

Western blotting showed that LPS reduced phosphorylated AKT and STAT3 and arginase‐1 while increasing iNOS. Treatments containing miR‐210‐3p reduced EFNA3 abundance, restored AKT and STAT3 phosphorylation, decreased iNOS and increased arginase‐1, with the largest changes generally observed for tFNAs‐miR‐210‐3p (Figure 3d,e). Total AKT and STAT3 were comparatively stable. The direction of EFNA3 regulation is consistent with the proposed inhibitory action of miR‐210‐3p on EFNA3; however, direct target validation and pathway perturbation are needed before describing the axis as definitively causal [10, 11, 12].

### 3.4 tFNAs‐miR‐210‐3p Improves Histological Repair and the Local Immune Microenvironment

**FIGURE 4:**
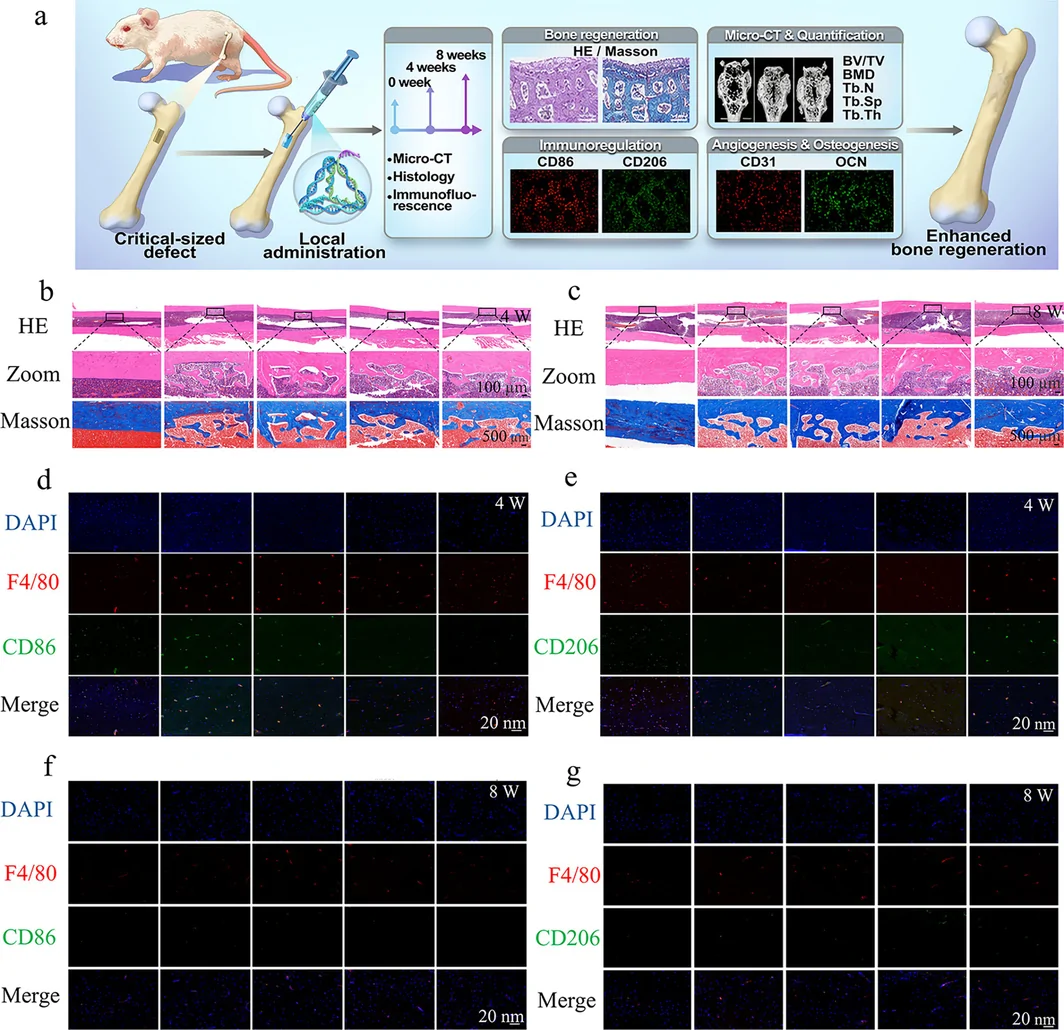
Histological repair and macrophage polarisation in rat femoral defects. (a) In vivo experimental design. (b,c) H&E and Masson's trichrome staining at 4 and 8 weeks. (d,e) F4/80 with CD86 or CD206 immunofluorescence at 4 weeks. (f,g) Corresponding immunofluorescence at 8 weeks. Groups are defined as in Figure 2.

The in vivo study evaluated local treatment in a rat critical‐sized femoral defect at 4 and 8 weeks (Figure 4a). H&E and Masson's trichrome staining showed progressive tissue repair across time, with denser and more continuous bone‐like tissue in the tFNAs‐miR‐210‐3p group (Figure 4b,c). Consistent with the natural progression of bone injury, macrophage polarisation exhibited obvious time‐dependent alterations: pro‐inflammatory M1 macrophages dominated the defect microenvironment in the early postoperative stage, while reparative M2 macrophages gradually accumulated as repair proceeded. At 4 weeks, the model group displayed a high CD86/F4/80 signal and a low CD206/F4/80 signal. tFNAs‐miR‐210‐3p significantly reduced the CD86/F4/80 ratio and increased the CD206/F4/80 ratio (Figures 4d,e and S1a), **e**xerting a prominent M2‐promoting effect at the early repair phase. At 8 weeks, the overall inflammatory response of all groups spontaneously subsided, leading to narrowed intergroup differences in macrophage phenotypes; nevertheless, tFNAs‐miR‐210‐3p retained the most favourable CD86/F4/80 and CD206/F4/80 profile (Figures 4f,g and S1b). These results suggest that early remodelling of the macrophage response contributes to subsequent repair [27, 28, 29].

### 3.5 tFNAs‐miR‐210‐3p Enhances Femoral Bone Regeneration, Vascularization and Osteogenesis

**FIGURE 5:**
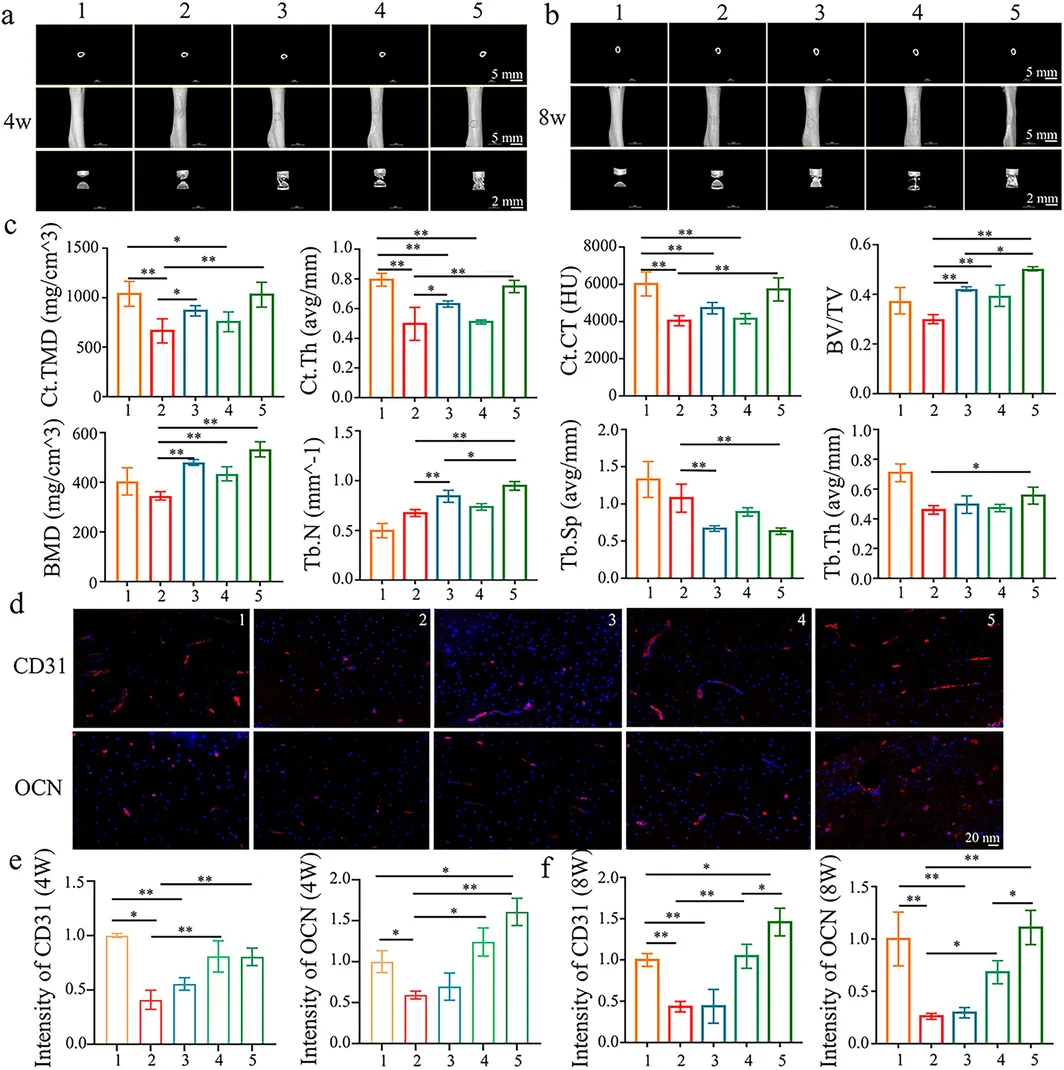
Micro‐CT evaluation, vascularization and osteogenesis after treatment of rat femoral defects. (a,b) Micro‐CT reconstructions at 4 and 8 weeks. (c) Quantification of trabecular and cortical bone parameters. (d) Representative CD31 and OCN immunofluorescence at 4 weeks. (e,f) Quantification of CD31 and OCN at 4 and 8 weeks. Groups are defined as in Figure 2. *p < 0.05 and **p < 0.01; confirm *n* and statistical comparisons.

Micro‐CT demonstrated greater defect bridging and structural restoration after tFNAs‐miR‐210‐3p treatment at both time points (Figure 5a,b). At 4 weeks, the nanocomposite improved BMD, BV/TV, Tb.N, Ct.TMD and Ct. CT relative to the model group, whereas several other trabecular and cortical parameters showed smaller or nonsignificant changes (Figures 5c and S1c). At 8 weeks, tFNAs‐miR‐210‐3p produced the broadest improvement across trabecular and cortical indices, including increased BMD, BV/TV, Tb.N, Tb.Th, Ct.TMD, Ct.CT and Ct.Th and reduced Tb.Sp [27, 28, 29].

CD31 and OCN immunofluorescence further linked structural repair with vascular and osteogenic responses (Figures 5d–f and S1d). At 4 weeks, free miR‐210‐3p and tFNAs‐miR‐210‐3p increased CD31 and OCN relative to the model, with the nanocomposite showing the strongest OCN response. At 8 weeks, tFNAs‐miR‐210‐3p produced the highest CD31 signal and increased OCN, while free miR‐210‐3p also improved OCN. The combined data indicate that efficient miR‐210‐3p delivery is particularly beneficial during early repair and supports sustained vascularized bone regeneration [25, 29].

### 3.6 Study Limitations and Translational Considerations

Several limitations should be addressed before translation. The present data do not establish the pharmacokinetics, biodistribution, degradation, long‐term safety or immunogenicity of tFNAs‐miR‐210‐3p. Direct binding of miR‐210‐3p to EFNA3 and the causal requirement for AKT/STAT3 signalling were not demonstrated by reporter, rescue or inhibitor studies in the supplied dataset. The durability of repair beyond 8 weeks and performance in larger animals also remain unknown. Future studies should quantify local retention and systemic exposure, establish dose–response relationships and include mechanistic perturbation and long‐term biomechanical testing [20, 21, 22].

## 4 Conclusions

A tFNA substantially improved intracellular delivery of miR‐210‐3p to macrophages and promoted a reparative phenotype associated with EFNA3 suppression and AKT/STAT3 phosphorylation. The nanocomposite enhanced angiogenic and osteogenic readouts in vitro and improved immune remodelling, vascularization, osteogenesis and structural repair in a rat critical‐sized femoral defect. These findings support tFNAs‐miR‐210‐3p as a promising immunomodulatory nucleic‐acid nanoplatform for vascularized bone regeneration.

## Author Contributions

Lei Hu performed most in vitro assays and the majority of animal surgeries, completed histological and micro‐CT imaging analyses, processed all experimental data and wrote the original manuscript. Kaixiao Xue aided in data statistics and manuscript editing. Jiahu Fang conceived the overall research design. Weitong Cui supervised the entire research program, secured study funding and revised the full manuscript. All authors read and approved the final submitted manuscript.

## Funding

This work was supported by the National Natural Science Fund for Young Scientists Fund Program C (82501707), Chengdu Municipal Medical Scientific Research Project (2026446), the China Postdoctoral Science Foundation (2025M771987), The Postdoctoral Fellowship Program (Grade C) of China Postdoctoral Science Foundation (GZC20251341), the Sichuan Province Innovative Talent Funding Project for Postdoctoral Fellows (BX202410), the Sichuan Provincial Natural Science Youth Foundation (2025ZNSFSC1649) and the Sichuan University Interdisciplinary Innovation Fund.

## Ethics Statement

This study was approved by the Ethics Committee of West China Hospital of Sichuan University (20260804001) and performed in accordance with the Declaration of Helsinki.

## Conflicts of Interest

The authors declare no conflicts of interest.

## Supporting information


**Table S1:** Base sequence of each ssDNA.
**Figure S1:** tFNAs‐miR210‐3p remodels macrophage polarisation and ameliorates bone loss in the LPS‐induced osteolytic mouse model.

## Data Availability

The data that support the findings of this study are available from the corresponding author upon reasonable request.
