# Supplementary material for: Tetrahedral Framework Nucleic Acid Delivery of miR‐210‐3p Promotes Femoral Bone Regeneration Through EFNA3/AKT/STAT3‐Mediated Immunomodulation
Source: Cell Prolif. 2026 Sep 1:e70277. Online ahead of print. doi: 10.1111/cpr.70277 (PMC13532375; doi:10.1111/cpr.70277)
Supplement: Supplementary file 1 [file CPR-9999-e70277-s001.docx]

**Supporting Information**

**Table S1** Base sequence of each ssDNA

| ssDNA (5′ → 3′) | Base sequence |
| --- | --- |
| S1 | ATTTATCACCCGCCATAGTAGACGTATCACCAGGCAGTTGAGACGAACATTCCTAAGTCTGAA |
| S2 | ACATGCGAGGGTCCAATACCGACGATTACAGCTTGCTACACGATTCAGACTTAGGAATGTTCG |
| S3 | ACTACTATGGCGGGTGATAAAACGTGTAGCAAGCTGTAATCGACGGGAAGAGCATGCCCATCC |
| S4 | ACGGTATTGGACCCTCGCATGACTCAACTGCCTGGTGATACGAGGATGGGCATGCTCTTCCCG |
| miR-210p | CUGUGCGUGUGACAGCGGCUGA |
| S3-miR-210p | ACTACTATGGCGGGTGATAAAACGTGTAGCAAGCTGTAATCGACGGGAAGAGCATGCCCATCC- CUGUGCGUGUGACAGCGGCUGA |

**Figure S1.** tFNAs-miR210-3p remodels macrophage polarization and ameliorates bone loss in the LPS-induced osteolytic mouse model. (a, b) Quantitative immunofluorescence intensity ratios of M1 marker CD86 and M2 marker CD206 normalized to pan-macrophage marker F4/80 in bone tissue at week 4 (a) and week 8 (b). Group 1: Control; Group 2: Model; Group 3: tFNAs treatment; Group 4: miR210-3p treatment; Group 5: tFNAs-miR210-3p treatment. (c) Quantitative micro-CT analysis of femoral bone microarchitectural parameters, including Ct.TMD, Ct. Th, Ct.CT, BV/TV, BMD, Tb.N, Tb.Sp and Tb.Th.(d) Representative immunofluorescence images of CD31 (vascular endothelial marker) and OCN (osteogenic marker) staining in bone sections, scale bar = 20 μm.

All data are presented as mean ± SD. *P < 0.05, **P < 0.01; N.S., not significant.
